# Development of 3D-Printed Cementitious Layered Model Rocks with Recycled Waste: A Study on Anisotropy

**DOI:** 10.3390/ma19102067

**Published:** 2026-05-15

**Authors:** Yongbo Hu, Yugao Wang, Zhenxing Wang, Shuying Wang, Jinsong Hu, Lehua Wang, Xiaoliang Xu

**Affiliations:** 1 Key Laboratory of Geological Hazards on Three Gorges Reservoir Area of Ministry of Education, China Three Gorges University, 8 Daxue Road, Yichang 443002, China; 2 Jilin Polytechnic of Water Resources and Electric Engineering, 6566 Juye Street, Changchun 130117, China; 3 China Three Gorges Corporation, 1 Liuhe Road, Wuhan 430010, China; 4 Xiantao Anjie Highway Maintenance Co., Ltd., 3 Hanjiang Road, Xiantao 433000, China

**Keywords:** layered rocks, anisotropy, 3D printing, failure mode, solid waste, CT scan

## Abstract

**Highlights:**

- Rock-like materials were developed using cement mortar and laboratory solid waste.
- The inherent anisotropy of the cement-based 3DP rocks was evaluated in three aspects.
- The internal structure of the 3DP rocks was observed using CT scanning.
- The mechanical properties of layered sandstones and 3DP layered rocks were compared.

**Abstract:**

Understanding the anisotropy in the physical and mechanical properties of layered rocks is essential for predicting and preventing instability in layered rock masses. However, in-situ sampling is often hindered by the difficulty of obtaining specimens with controlled bedding orientations. Cement-based 3D printing (3DP) offers an efficient approach for fabricating rock analogues, yet the inherent anisotropy induced by the layer-by-layer deposition process has not been well characterized, hindering its broader application. The objectives of this study are (i) to systematically evaluate the intrinsic anisotropy of cement-based 3DP rocks and (ii) to compare the mechanical anisotropy and failure modes of 3DP layered rocks with those of natural layered sandstone. The key findings are as follows: (1) The uniaxial compressive strength (UCS), P-wave velocity, and computed tomography (CT) number of the 3DP rock vary by less than 6% among the X-, Y-, and Z-directions, indicating lower intrinsic anisotropy compared to typical sandstones and several other natural rocks. (2) The UCS, elastic modulus, and secant modulus of the 3DP layered rocks all decrease initially and then increase with bedding dip angle, reaching a minimum at 60°. (3) The main fracture characteristics of the 3DP layered rocks are similar to those of layered sandstone; notably, the 3DP layered soft rock exhibits the most pronounced shear failure features. This study quantifies the low intrinsic anisotropy of cement-based 3DP rocks and validates their similarity to natural layered sandstone in both mechanical anisotropy and failure modes. It thereby provides a reliable, reproducible basis for physical modeling of layered rock masses using 3DP, offering a new approach for laboratory-scale investigations of layered rocks.

## 1. Introduction

In geological, hydropower, and mining engineering projects, layered rocks with different bedding dip angles are inevitably encountered. Due to the presence of weak bedding planes, these rocks exhibit pronounced anisotropy [1,2,3]. Clarifying their physical and mechanical properties is therefore essential for ensuring the safety and stability of related engineering activities. However, field sampling of layered rocks remains challenging, and the resulting test specimens often show considerable variability [4,5]. Cement (gypsum)-based 3DP technology offers distinct advantages such as mold-free fabrication and high specimen preparation efficiency. Notably, the layer-by-layer deposition process is highly consistent with the natural bedding structure of layered rocks, making this technology particularly suitable for producing layered rock models [6,7,8,9].

Recently, cement (gypsum)-based 3DP technology has been increasingly applied in rock mechanics [10,11,12,13]. Feng et al. [14] identified four key factors affecting the quality of cement-based 3DP specimens and proposed an efficient method for printing large-scale geological physical models with controllable internal structures. Wu et al. [15] investigated effective approaches for improving the strength and stiffness of gypsum-based 3DP soft rock specimens. Based on similarity theory, Mei et al. [16] and Mei et al. [17] simulated the entire rockburst process associated with tunnel faults using cement-based 3DP technology. Feng et al. [18] and Wang et al. [19] investigated the size effect of a 3DP cavern model and verified the rationality of combining the 3DP model with an engineering prototype. Wang et al. [20] focused on the size effect of the mechanical parameters of a cement-based 3DP fractured rock mass model. While these studies provide valuable references, none of them adequately considered the anisotropy resulting from the layer-by-layer fabrication process in cement (gypsum)-based 3DP. There remains a lack of understanding and evaluation regarding whether the inherent anisotropy of 3DP models affects test results and, if so, to what extent.

Previous studies have investigated the physical and mechanical properties of 3DP layered rock-like materials using CT scanning, scanning electron microscopy (SEM), uniaxial compression tests (UCTs), and triaxial compression tests [21,22,23,24]. Xu et al. [25] investigated the influence of printing layer thickness on the physical and mechanical properties of 3DP rock specimens. Santiago et al. [26] explored the physical and mechanical properties of 3DP layered coal rocks; however, the bedding dip angles of the specimens were limited to horizontal and vertical orientations. He et al. [27] and Shao et al. [28] printed rock-like specimens with different bedding dip angles using gypsum as a raw material and analyzed the resulting mechanical properties and failure modes. Most of the aforementioned studies used gypsum as the printing material. However, gypsum is characterized by relatively low strength and stiffness, making it suitable only for simulating soft rock. In contrast, cement-based materials exhibit superior mechanical properties and can meet the mechanical parameter requirements of typical hard rocks. Overall, experimental research on the anisotropic physical and mechanical properties of cement-based 3DP layered hard rocks remains limited and requires further investigation. Comparative studies on the physical and mechanical properties of 3DP layered hard rocks, 3DP layered soft rocks, and natural layered rocks also warrant additional investigation. Furthermore, few studies have comprehensively addressed the issues of solid waste disposal and reuse in laboratory settings.

Accordingly, the present study focuses on layered sandstone (hard rock) and layered mudstone (soft rock) collected from the Three Gorges Reservoir area in China. 3DP hard rock (3DP-HR), 3DP layered hard rock (3DP-LHR), and 3DP layered soft rock (3DP-LSR) models were fabricated using 3DP technology. From the perspectives of mechanical behavior, acoustic characteristics, and internal structure, a comprehensive quantitative assessment of the inherent anisotropy of cement-based 3DP rock materials was conducted through UCTs, P-wave velocity measurements, and CT scanning. A comparative analysis was then performed to examine the variations in the key mechanical parameters of 3DP-LHR, 3DP-LSR, and natural layered sandstone with bedding dip angle, as well as the similarities and differences in their failure modes. The research findings demonstrate the feasibility of using cement-based 3DP technology, with solid wastes as raw materials, to fabricate rock and layered rock mass models. They also provide new insights for the stability control of layered rock masses and the resource utilization of solid wastes such as rosin and barite powder.

## 2. Model Creation and Sampling

**Figure 1:**
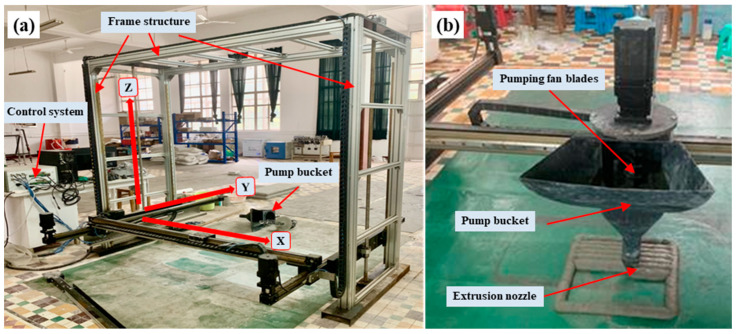
Concrete (mortar) 3D printer: (**a**) control system and frame structure; (**b**) 3DP pump bucket.

The concrete (mortar) 3DP machine (Hangzhou Jianyan Technology Co., Ltd., Zhejiang, China) used in the experiment is shown in Figure 1a. The printer primarily consists of a support frame, three-dimensional moving axes along the X-, Y-, and Z-directions, a control system, and a pump bucket, as shown in Figure 1b. Before printing, the pump bucket was mounted on the X-axis. The diameter of the pump bucket extruder nozzle was 20 mm. The horizontal movement speed of the pump bucket during printing was 50 mm/s, and the vertical lifting speed was 5 mm/s. The mechanical control precision of the printer was within 0.1 mm.

### 2.1. Testing Material

According to previous studies [29,30,31], the UCS of certain sandstones in the Three Gorges Reservoir area ranges from approximately 40 to 60 MPa, while their elastic modulus ranges from approximately 5 to 13 GPa. Similarly, the UCS of some mudstones ranges from approximately 10 to 20 MPa, and their elastic modulus ranges from approximately 1.5 to 3.5 GPa [32,33,34]. The development of 3DP simulated materials for hard rock (sandstone-like) and soft rock (mudstone-like) is based on these two primary mechanical parameters: UCS and elastic modulus.

#### 2.1.1. Hard Rock Simulation Material

**Figure 2:**
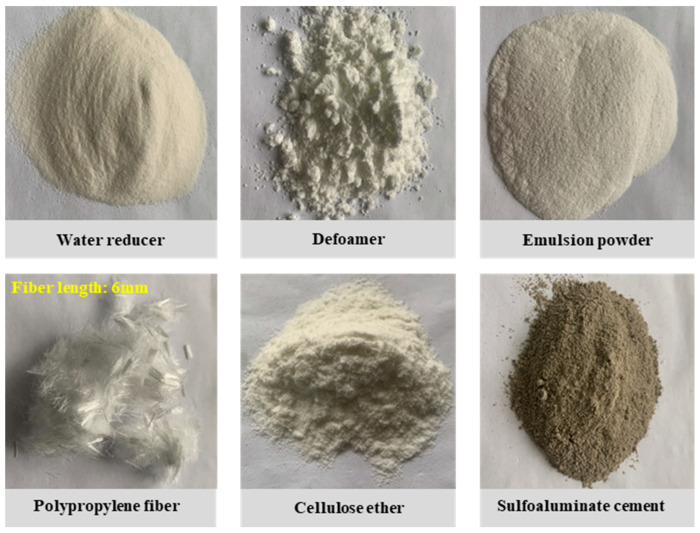
Photographs of the admixtures and supplementary materials added to the cement mortar.

Conventional cement mortar cannot meet the thixotropy, pumpability, and printability requirements of cement-based 3DP processes. Therefore, additives and supplementary materials must be incorporated to modify its rheological and mechanical properties (Figure 2). Based on previous studies, the dosages of admixtures and supplementary materials were iteratively adjusted [35,36,37]. After repeated testing, a set of mix proportions for the simulated hard rock material was obtained, which allowed for stable printing and closely matched the key mechanical parameters of hard rock, such as UCS and elastic modulus, as listed in Table 1.

**Figure 3:**
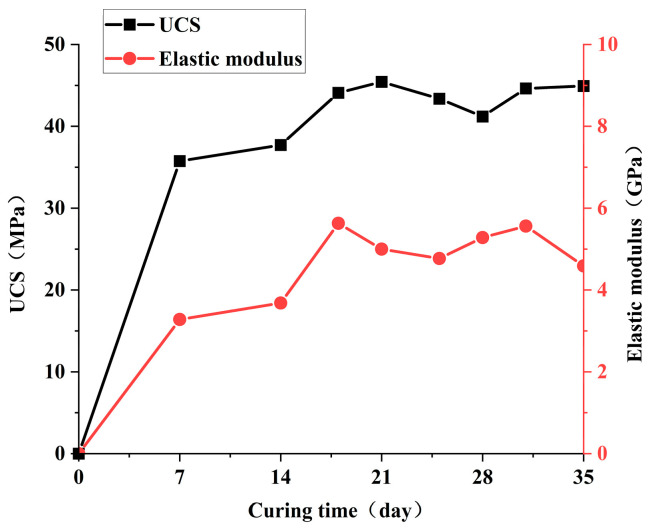
UCS and elastic modulus of the hard rock-simulating material as a function of curing time.

The variations in the UCS and elastic modulus of the simulated hard rock material with curing time are shown in Figure 3. The UCS of the cast specimens gradually stabilized at approximately 45 MPa after 18 days of curing. Similarly, the elastic modulus tended to stabilize and fluctuated around 5 GPa. Therefore, the subsequent curing period for the hard rock models was set to no less than 18 days.

As shown in Table 2, the primary mechanical properties were measured from specimens extracted after the 3DP-HR model had been cured for 18 days. The specimens were obtained by drilling in the vertically downward direction. The UCS and elastic modulus values of sandstone and 3DP-HR were found to be largely comparable.

#### 2.1.2. Soft Rock Simulation Material

Using the mix proportion of the established hard rock-simulating material as a basis, a soft rock-simulating material was developed by incorporating laboratory solid waste. The mass fractions of the components in the solid waste were as follows: quartz sand 23.9%, barite powder 71.5%, gypsum 4%, and rosin 0.6%. Prior to use, the solid waste was crushed and sieved to a particle size range of 30–300 mesh (0.05–0.6 mm).

In this simulating material, quartz sand and river sand together serve as aggregates. Quartz sand particles have relatively rough surfaces, which can form effective mechanical interlocking with the cement paste, thereby helping to reduce microcracks generated by drying shrinkage of the mortar. Owing to its higher hardness and strength compared with river sand, quartz sand contributes to a moderate improvement in the strength and elastic modulus of the mortar.

Barite powder has a high density of approximately 4.0–4.5 g/cm^3^ and, when incorporated, can significantly increase the plastic viscosity and density of the mortar. The increased density helps the printed material resist the self-weight pressure imposed by the overlying layers, thereby reducing the vertical deformation of the 3DP structure. Barite powder can also act as a fine filler, occupying the voids between cement particles and aggregates and thereby providing a physical densification effect. However, excessive incorporation of barite powder may dilute the cementitious binder phase, leading to a decline in mechanical properties.

Gypsum reacts with tricalcium aluminate in cement to form ettringite. The formed ettringite can coat the surfaces of cement particles, thereby prolonging the initial setting time and open time of the 3D-printable mortar, i.e., the printability window. Meanwhile, ettringite formation is accompanied by slight expansion, which can partially compensate for the deformation of the mortar induced by drying shrinkage. An appropriate dosage of gypsum, approximately 3–5% by mass of cement, can enhance the early-age strength, whereas excessive gypsum incorporation may significantly reduce the long-term strength.

Rosin is a natural surfactant; its hydrophobic groups can be directionally adsorbed onto the surfaces of cement particles or at the gas–liquid interface, thereby lowering the surface tension of water. During mortar mixing, rosin can entrain a small amount of fine, closed air bubbles. These bubbles enhance the extrudability of the printable material and reduce pumping pressure; however, they also increase the total porosity and negatively affect the UCS and elastic modulus.

The cementitious capacity of the solid waste is substantially lower than that of cement. With the contents of cement and river sand kept constant, increasing the solid waste content weakens the cementitious behavior and mechanical properties of the composite material. Through repeated experiments, two mix proportions of the soft rock-simulating material with different solid waste contents were determined, as presented in Table 3. The mixture with a higher solid waste content is referred to as the degraded soft rock-simulating material, which is primarily used for comparison with the regular soft rock-simulating material.

The proportion of sulfoaluminate cement, which is characterized by rapid hardening and high early-age strength, was kept unchanged in the soft rock-simulating material relative to that in the hard rock-simulating material. The strength development period associated with gypsum and barite powder is generally shorter than 18 days. Meanwhile, the proportion of early-strength water reducer was higher in both the soft rock-simulating material and the degraded soft rock-simulating material. Consequently, the soft rock model required a curing period of only 18 days. As shown in Table 4, the UCS and elastic modulus of mudstone and 3DP-SR are generally comparable. In contrast, the mechanical properties of 3DP-DSR are significantly lower than those of 3DP-SR.

### 2.2. Model Printing and Sampling

**Figure 4:**
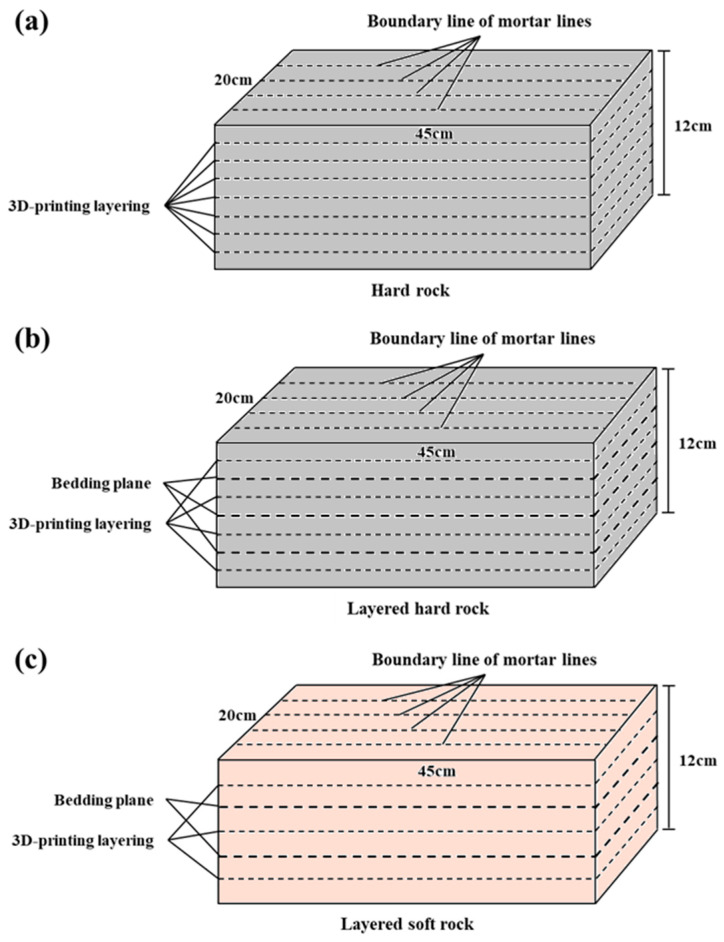
Schematic diagrams of the 3DP rock models: (**a**) 3DP hard rock (3DP-HR) model; (**b**) 3DP layered hard rock (3DP-LHR) model; (**c**) 3DP layered soft rock (3DP-LSR) model.

For the convenience of printing, handling, curing, and specimen extraction, the rock model dimensions were designed as follows: 45 cm in length, 20 cm in width, and no less than 12 cm in height. The layer height of the hard rock-simulating material ranges from 1.5 to 1.6 cm, whereas that of the soft rock and degraded soft rock-simulating materials ranges from 1.9 to 2.0 cm. The soft rock-simulating material exhibits higher fluidity due to its higher water–cement ratio. Under the same printing conditions, more mortar is extruded from the nozzle, resulting in a greater layer thickness than that of the hard rock-simulating material. As shown in Figure 4a, the 3DP-HR model does not contain internal bedding planes. The 3DP-LHR model and the 3DP-LSR model are shown in Figure 4b,c, respectively, and both contain internal bedding planes. In Figure 4, the 3DP layering is generated by the layer-by-layer deposition process, in which successive mortar layers are automatically bonded together. By contrast, the bedding planes were intentionally introduced, and the corresponding simulation procedure is described below.

**Figure 5:**
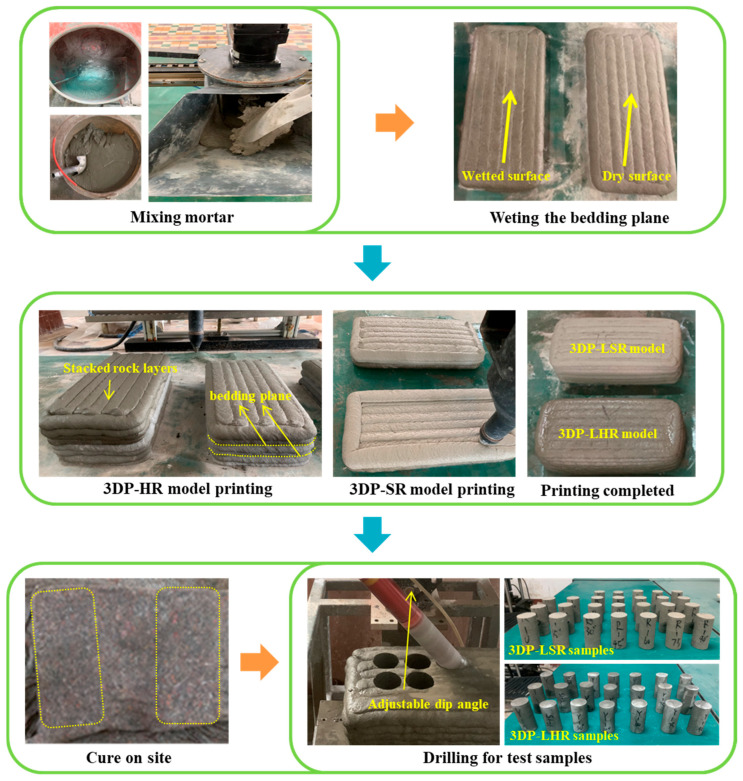
Fabrication, curing, and sampling procedures for the 3DP layered rock model.

Figure 5 shows the fabrication procedure for the 3DP layered rock models. During mortar mixing, the dry components were first mixed for 1 min to ensure uniform distribution, followed by the addition of water and mixing for approximately 2.5 min. The bedding planes were simulated by introducing a 24 h time interval between successive printed layers. After the first layer was completed, the model was left undisturbed for 24 h. Before printing the second layer, a moderate amount of water mist was evenly sprayed onto the first layer to moisten the surface without producing visible water accumulation. After model printing was completed, the specimens were precured for 24 h and then transferred to the curing environment. The curing location was maintained at a temperature of 20 ± 2 °C and a relative humidity consistently greater than 95%. After curing, specimens with bedding dip angles of 0°, 15°, 30°, 45°, 60°, 75°, and 90° were drilled and prepared. For each dip angle, no fewer than three specimens were prepared to ensure at least three replicates in each test group. The bedding dip angle is defined as the angle between the bedding plane and the horizontal plane. After cutting and grinding to obtain flat and parallel end surfaces, 3DP-LHR and 3DP-LSR specimens with a height of 100 mm and a diameter of 49 mm were obtained. The tolerances of specimen height and diameter were controlled within 0.3 mm, and the surface irregularity at both ends was controlled within 0.05 mm. Direct shear tests revealed that the cohesion of the bedding planes in 3DP-LHR was 0.55 MPa, with an internal friction angle of 50.23°. For 3DP-LSR, the cohesion was 0.49 MPa, with an internal friction angle of 47.67°.

**Figure 6:**
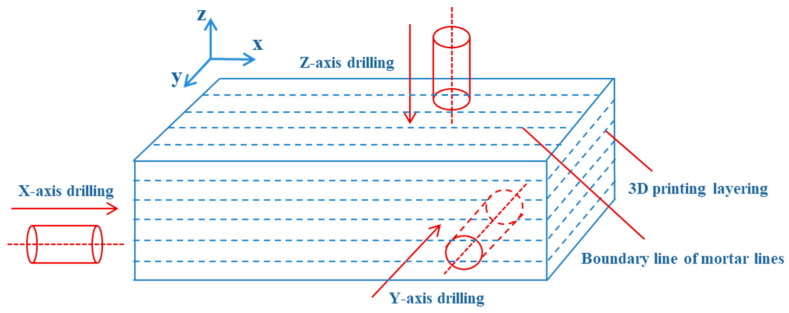
Schematic diagram of specimen drilling along the X-, Y-, and Z-directions in the 3DP-HR model (excluding bedding planes).

Before conducting experiments on 3DP layered rocks, it is essential to characterize the physical properties and internal structural features of cement-based 3DP rock. Taking 3DP-HR (Figure 4a) as an example, Figure 6 shows that 3DP-HR specimens were drilled along the X-, Y-, and Z-directions for subsequent physical and mechanical testing.

## 3. Physical Properties Analysis

### 3.1. Density and Porosity

The density and porosity of different rock-like simulating materials were measured using a high-precision electronic scale, a vernier caliper, and a MacroMR12-150H-I nuclear magnetic resonance imaging analyzer (Suzhou Niumag Analytical Instrument Corporation, Jiangsu, China). For each group, three replicate tests were performed, and the median value of the three measurements was adopted, as listed in Table 5. The air-dry density, saturated density, and porosity of 3DP-HR are all lower than those of 3DP-SR and 3DP-DSR. This is mainly because the solid waste incorporated into 3DP-SR and 3DP-DSR contains high-density barite powder, thereby resulting in higher densities than that of 3DP-HR. In addition, the solid waste also contains rosin. Rosin has emulsifying and air-entraining effects in cement mortar, which contribute to significantly higher porosity in 3DP-SR and 3DP-DSR than in 3DP-HR.

**Figure 7:**
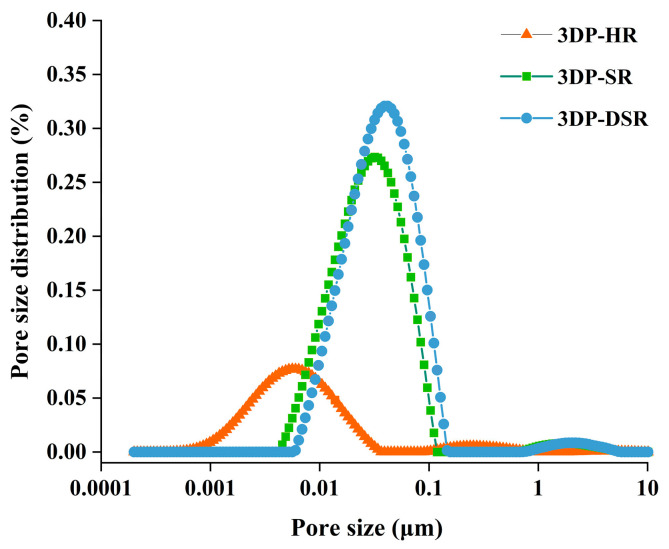
Pore size distribution of 3DP-HR, 3DP-SR, and 3DP-DSR specimens.

The pore size distributions of 3DP-HR, 3DP-SR, and 3DP-DSR are shown in Figure 7, where the x-direction is plotted on a natural logarithmic scale. The pore sizes of the rock-like simulating materials were mostly in the range of 0.001–0.1 μm, showing an approximately normal distribution. The maximum pore size of 3DP-HR was significantly smaller than those of 3DP-SR and 3DP-DSR, and its pore size distribution was more uniform. Under the influence of rosin, 3DP-SR and 3DP-DSR exhibited larger pore sizes and a more concentrated distribution. This indicates that rosin can increase both the porosity and maximum pore size of cement-based materials.

### 3.2. Relationship Between P-Wave Velocity and Other Physical and Mechanical Parameters

Sound waves are elastic waves. When rock is treated as an elastic medium, the propagation of sound waves through the rock mass follows the same principles as the propagation of elastic waves. According to the Lamé equations of motion in elasticity theory:

(1) $$\begin{array}{l} (\lambda +G)\frac{\partial \theta }{\partial x}+G\nabla^{2}u=\rho \frac{\partial^{2}u}{\partial t^{2}}\\ (\lambda +G)\frac{\partial \theta }{\partial y}+G\nabla^{2}v=\rho \frac{\partial^{2}v}{\partial t^{2}}\\ (\lambda +G)\frac{\partial \theta }{\partial z}+G\nabla^{2}w=\rho \frac{\partial^{2}w}{\partial t^{2}} \end{array}$$

where

(2) $$\lambda =\frac{\upsilon E}{\left(1+\upsilon )(1-2\upsilon \right)}$$

(3) $$G=\frac{E}{2(1+\upsilon )}$$

(4) $$\theta =\varepsilon_{x}+\varepsilon_{y}+\varepsilon_{z}=\frac{\partial u}{\partial x}+\frac{\partial v}{\partial y}+\frac{\partial w}{\partial z}$$

(5) $$\nabla^{2}=(\frac{\partial^{2}}{\partial x^{2}}+\frac{\partial^{2}}{\partial y^{2}}+\frac{\partial^{2}}{\partial z^{2}})$$

Here, λ and G are the Lamé coefficients, υ is Poisson’s ratio, θ is the bulk modulus, $\nabla^{2}$ is the Laplacian, ρ is the density, E is the elastic modulus, and u, v, and w are the displacements of the particle along the X-, Y-, and Z-axes in the rectangular coordinate system, respectively.

Because the propagation direction of the P-wave is consistent with the vibration direction of the particle, it can be assumed that u=u(x,t), v=0, and w=0. Substituting these into Equation (1) yields:

(6) $$(\lambda +2G)\frac{\partial^{2}u}{\partial x^{2}}=\rho \frac{\partial^{2}u}{\partial t^{2}}$$

Equation (6) can also be written as

(7) $$\frac{\partial^{2}u}{\partial t^{2}}=V_{p}^{2}\frac{\partial^{2}u}{\partial x^{2}}$$

(8) $$V_{p}=\sqrt{\frac{\lambda +2G}{\rho }}=\sqrt{\frac{E(1-\upsilon )}{\rho (1+\upsilon )(1-2\upsilon )}}$$

where $V_{p}$ is the longitudinal wave velocity, ρ is the density, and E is the elastic modulus.

**Figure 8:**
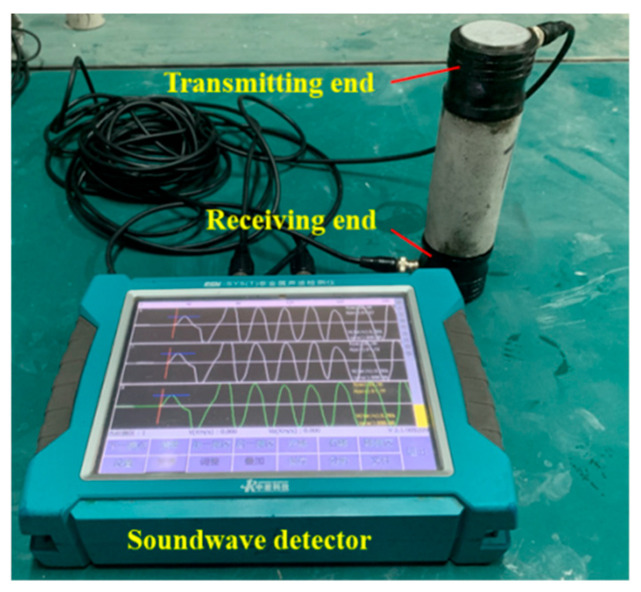
Testing the P-wave velocity of the specimen using the RSM-SY5(T) non-metallic acoustic testing instrument.

**Figure 9:**
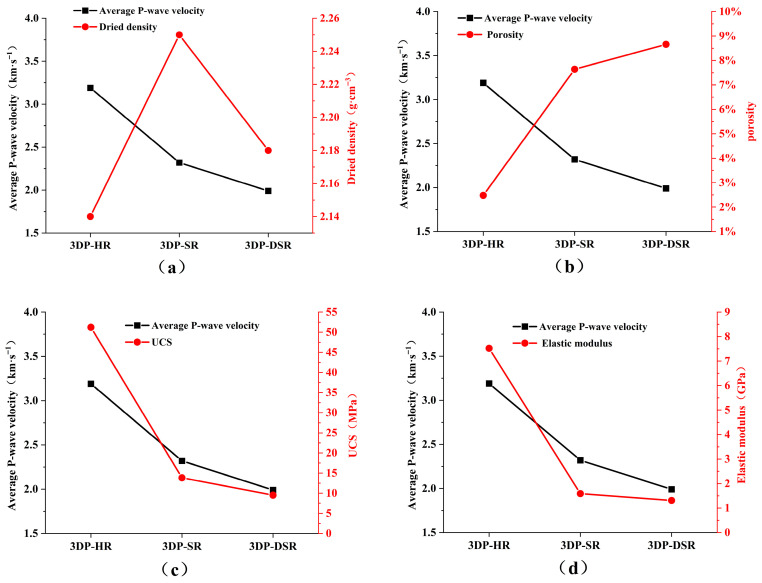
Comparison of P-wave velocities for 3DP-HR, 3DP-SR, and 3DP-DSR specimens with the corresponding (**a**) dry density, (**b**) porosity, (**c**) uniaxial compressive strength, and (**d**) elastic modulus.

Equation (8) shows that the P-wave velocity depends on the density and the Lamé coefficients λ and G of the medium. In rock mechanics, the Lamé coefficients λ and G are referred to as the bulk modulus and the shear modulus, respectively. This indicates that P-wave propagation in a medium is jointly influenced by both compression and shear effects. The P-wave velocity is also related to the elastic modulus and Poisson’s ratio of the medium. Therefore, the P-wave velocity of rock can, to some extent, reflect its integrity, density, and internal structural characteristics. P-wave velocity tests were conducted on the 3DP rocks using the RSM-SY5(T) non-metallic acoustic testing instrument (Wuhan Sinorock Technology Co., Ltd., Wuhan, China), as shown in Figure 8. For each group, no fewer than three specimens were prepared, and no fewer than three replicate measurements were performed. Data points with a relative deviation from the group mean exceeding ±10% were excluded. Figure 9 compares the P-wave velocities of 3DP-HR, 3DP-SR, and 3DP-DSR with their corresponding physical and mechanical parameters. The specimens were drilled along the Z-direction. Although the incorporated barite powder has a high density, the foaming effect of rosin increases the porosity, which may explain why the air-dry density of 3DP-DSR was lower than that of 3DP-SR. Generally, the lower the porosity of a natural rock, the denser it is; accordingly, denser rocks usually exhibit higher P-wave velocities and greater strength. For 3DP rocks, lower porosity also leads to a higher average P-wave velocity and greater average UCS. These three parameters are mutually consistent, and the trend is consistent with that observed in natural rocks.

### 3.3. Internal Structural Characteristics of 3DP Rocks

CT scanning technology (Royal Dutch Philips Electronics Ltd., The Netherlands) was used to obtain grayscale images of different cross-sectional and longitudinal slices of the specimens. Adjacent sections were spaced 2 mm apart. The grayscale image consists of pixels with varying shades of gray, where lighter shades indicate higher density. Such grayscale images reflect the internal structural characteristics of the rock.

**Figure 10:**
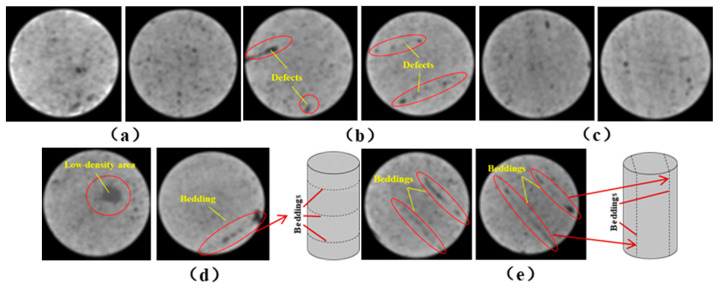
Representative cross-sectional grayscale images of 3DP hard rock specimens: (**a**) Z-direction; (**b**) X-direction; (**c**) Y-direction; (**d**) bedding dip angle of 0°; (**e**) bedding dip angle of 90°.

One randomly selected specimen of each type was subjected to CT scanning. From the series of cross-sectional grayscale images obtained for each specimen, two representative cross-sectional images and one longitudinal image were selected, as shown in Figure 10. The lighter gray areas represent the specimen matrix, whereas the darker areas correspond to internal defects. The following observations can be derived from Figure 10: (1) Among the specimens tested along the X-, Y-, and Z-directions, only the X-direction specimen exhibited a defect distribution with an approximately linear configuration. This finding suggests the presence of partial weak zones and voids at the joints between adjacent mortar strips. (2) In the cross-section of the 0° 3DP-LHR specimen, the linear defects correspond to the undulations along the bedding planes. The interparticle bonding at the bedding planes is less compact than that in the matrix, and more pores are present; consequently, dark low-density areas are more likely to occur near the bedding planes. (3) In both representative cross-sections of the 90° 3DP-LHR specimen, two nearly linear defects are observed, and the bedding-related defect characteristics are particularly prominent.

**Figure 11:**
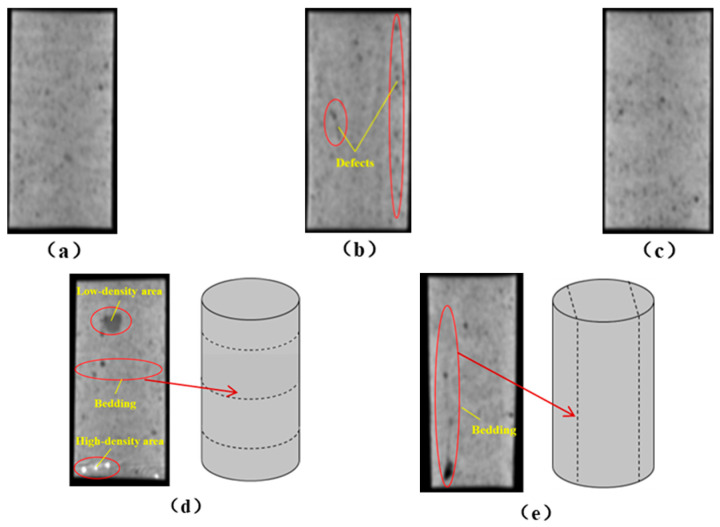
Representative longitudinal-section grayscale images of 3DP hard rock specimens: (**a**) Z-direction; (**b**) X-direction; (**c**) Y-direction; (**d**) bedding dip angle of 0°; (**e**) bedding dip angle of 90°.

The grayscale images of representative longitudinal sections of the five specimens are shown in Figure 11. The following observations can be made from Figure 11: (1) The X-direction specimen exhibits longitudinal linear defects with a relatively regular arrangement, which corresponds to the observation in Figure 10b. The internal defect positions in the specimens drilled along the Y- and Z-axes show no obvious distribution pattern, which is consistent with Figure 10a,c. (2) The low-density area in Figure 11d corresponds to that in Figure 10d. This low-density area may be a bubble-rich matrix formed near the bedding plane or an undispersed agglomeration of cementitious material. The high-density area appearing at the bottom of the specimen is likely attributable to relatively large sand or gravel particles. (3) The longitudinal linear defects in the 90° 3DP-LHR specimen correspond to those observed in Figure 10e. As shown in Figure 10 and Figure 11, the bedding-plane-related defects in the 3DP layered rocks are evident, fully demonstrating the feasibility of the proposed fabrication method for layered rock models. Both 3DP rock and 3DP layered rock contain a small number of structural defects induced by the printing process. Whether these inherent structural defects affect the physical and mechanical test results will be discussed in the following section.

## 4. Anisotropy of Cement-Based 3DP Rocks

To fully understand the inherent anisotropy of cement-based 3DP rock, the anisotropy of 3DP-HR was evaluated from three aspects, namely UCS, P-wave velocity, and CT number.

### 4.1. Anisotropy of Uniaxial Compressive Strength

Selected fundamental mechanical parameters of the 3DP rock specimens were obtained through UCTs, Brazilian splitting tests, and direct shear tests, as detailed in Table 6. For each test group, no fewer than three replicate tests were conducted.

As shown in Table 6, the average UCS values of the 3DP-HR specimens drilled along the X-, Y-, and Z-directions are 53.53 MPa, 56.38 MPa, and 51.21 MPa, respectively, with the Z-direction specimens exhibiting the lowest UCS. This can be attributed to the presence of defects at the interlayer interfaces within the 3DP rock model; the specimens oriented along the Z-direction contain the largest number of such interfaces, thus yielding the lowest UCS. The standard deviation of the mean UCS values among the three directions is only 2.59 MPa, and the UCS variation within each direction does not exceed 6%, which falls within the generally accepted variation range of ±10% for rock UCS tests. Consequently, the inherent strength anisotropy of 3DP rock is relatively weak and exerts only a limited influence on the test results. The UCS, elastic modulus, tensile strength, and cohesion of 3DP-HR, 3DP-SR, and 3DP-DSR decrease in sequence. This is attributed to the gradually increasing content of solid waste, which leads to a progressive weakening of the cementation between particles in the rock-like simulating material.

### 4.2. Anisotropy of P-Wave Velocity

**Figure 12:**
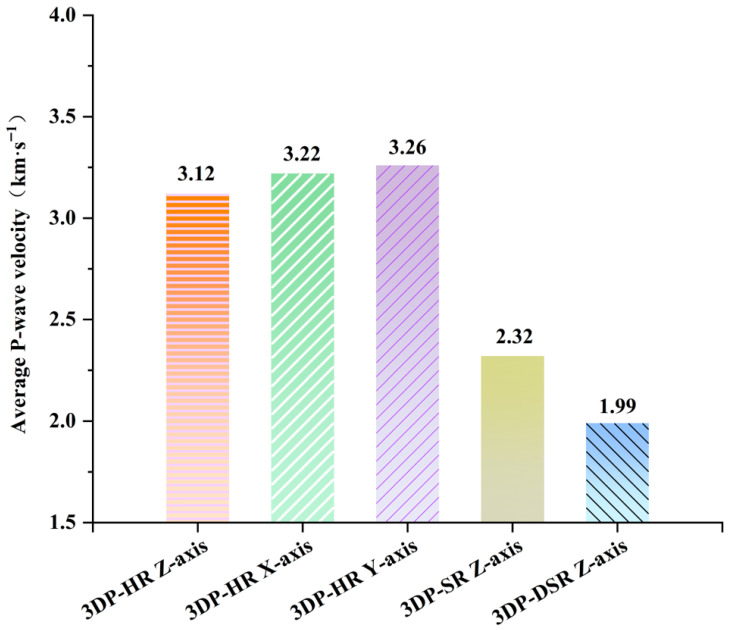
Average P-wave velocity of 3DP-HR, 3DP-SR, and 3DP-DSR specimens.

Figure 12 shows the P-wave velocity test results for 3DP-HR, 3DP-SR, and 3DP-DSR. The average P-wave velocities of the 3DP-HR specimens drilled along the X-, Y-, and Z-directions are relatively close, with a standard deviation of 0.072 km/s and a variation range of less than 5%. In ref. [38], P-wave velocity tests were conducted on a large number of natural rock specimens, including sandstone and granite, and the obtained P-wave velocity fluctuations all exceeded 10%. This indicates that the P-wave velocity anisotropy of 3DP-HR is less pronounced than that of natural rocks. For the drilled specimens, the P-wave velocity is the highest along the Y-direction and the lowest along the Z-direction, which is consistent with the pattern observed for UCS. This indicates that the 3DP rock is relatively denser and contains fewer defects along the Y-direction. The P-wave velocities of 3DP-HR ranged from 3.12 to 3.26 km/s. The average P-wave velocities of 3DP-SR and 3DP-DSR were 2.32 km/s and 1.99 km/s, respectively. These values all fall within the measured P-wave velocity range of sandstone and mudstone in the Three Gorges Reservoir area of China [34,39].

**Figure 13:**
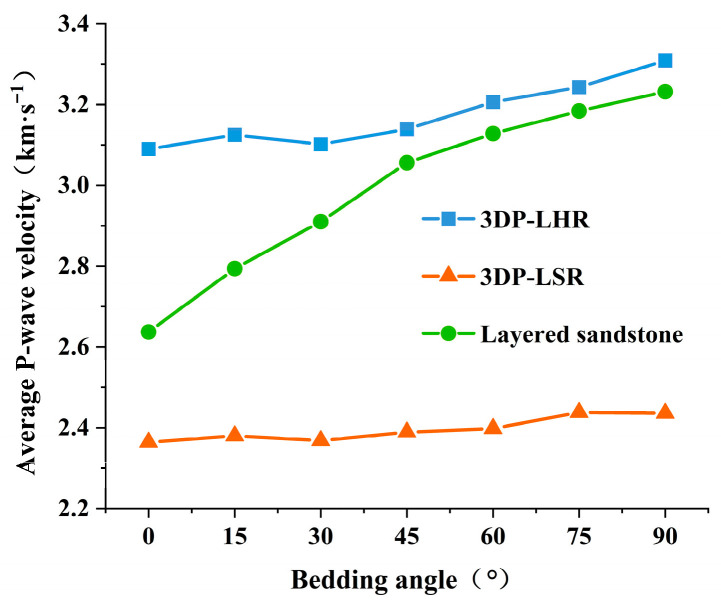
Variation in the average P-wave velocity of 3DP-LHR, 3DP-LSR, and layered sandstones specimens with bedding dip angles.

As shown in Figure 13, the average P-wave velocities of both 3DP-LHR and 3DP-LSR increase with increasing bedding dip angle, which is consistent with the trend reported for natural layered sandstone [39]. Layered rock consists of a dense, high-velocity matrix and relatively loose, low-velocity bedding planes. The layered sandstone specimens contain more bedding planes than the 3DP-LHR specimens, resulting in an overall lower P-wave velocity and a more pronounced dip-angle dependence. The P-wave velocity of 3DP-LHR varies only slightly within the range of 0–30°, probably because the surface undulation of its bedding planes partially offsets the influence of changes in bedding dip angle.

As the bedding dip angle increases from 0° to 90°, the angle between the P-wave propagation direction and the bedding planes gradually decreases, thereby weakening wave reflection at the interfaces and reducing energy attenuation. Meanwhile, the number of weak bedding planes intersected by the wave path decreases, reducing the proportion of the low-velocity medium along the propagation path. Consequently, the travel time shortens, and the wave velocity increases continuously. Ultimately, the relative orientation between the propagation direction and the aligned bedding planes determines the proportional propagation paths through the matrix and bedding planes, thus governing the macroscopic wave velocity.

### 4.3. Characteristics of CT Number Changes

**Figure 14:**
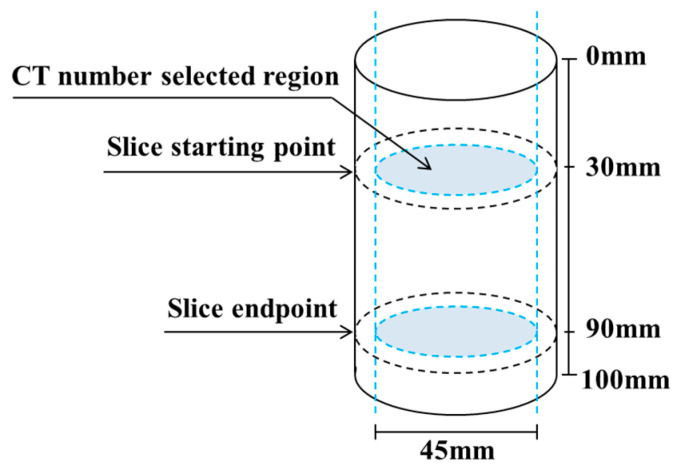
Schematic diagram of specimen slice positions and the selected region for CT number measurement.

The pixel value at any point in a grayscale image obtained from CT scanning can be numerically expressed. The mean value of all pixels within a specific region is defined as the CT number. The CT number is directly proportional to density, thereby enabling a quantitative evaluation of the local density of the specimen to some extent [40]. The starting and ending positions of the CT scanning sections of the specimen are shown in Figure 14. The regions used for CT number extraction on different cross-sections of the same specimen were kept identical, each with a diameter of 45 mm.

**Figure 15:**
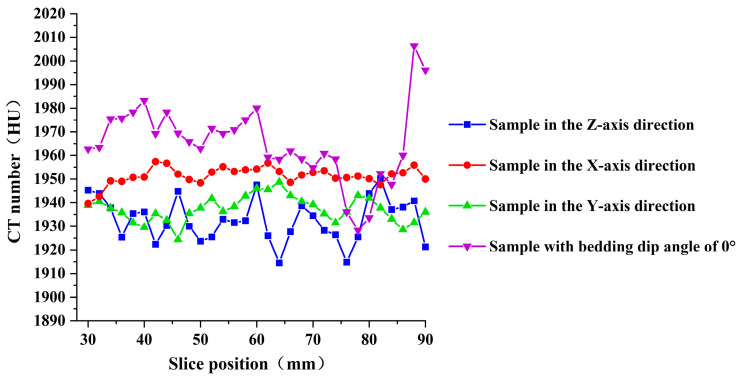
CT numbers of four 3DP hard rock specimens at different slice positions.

Figure 15 shows the variation in CT numbers with slice position for the four types of specimens. The following observations can be made: (1) The average CT numbers of the four specimen types fluctuate irregularly with slice position. The corresponding standard deviations are 12.06, 8.42, 7.88, and 16.6, respectively, and the CT number variation ranges are all within 5%, indicating that the internal composition of the specimens is generally homogeneous. (2) The specimen with a bedding dip angle of 0° exhibits the largest standard deviation, which is attributed to the presence of bedding planes. (3) Among the specimens drilled along the X-, Y-, and Z-directions, the Z-direction specimen shows the largest standard deviation and the lowest average CT number, suggesting that it contains the greatest number of internal defects and has the loosest internal structure. This is consistent with the UCS and P-wave velocity results. For the intact specimens drilled along the X-, Y-, and Z-directions, the variation in the average CT number is only within 3%. As reported in ref. [40], the CT numbers of argillaceous marl, siltstone, and fine sandstone are close to those of the 3DP rocks, yet their CT number variations exceed 3%. This further confirms, from the perspective of CT numbers, the good homogeneity and weak anisotropy of the 3DP rocks.

Based on the results of UCT, P-wave velocity measurements, and CT scanning, the anisotropy of the 3DP rock is not pronounced, and its influence on the experimental results is weaker than that of natural rocks. Therefore, it is feasible to use cement-based 3DP technology to fabricate layered rock models for physical and mechanical testing.

## 5. Anisotropy of Mechanical Properties and Failure Modes

### 5.1. Stress–Strain Curve

UCTs were conducted using the RMT-150C rock mechanics testing system (Wuhan Institute of Rock and Soil Mechanics, Chinese Academy of Sciences, Wuhan, China). The apparatus employed a force-controlled loading procedure with a loading rate of 0.5 kN/s. No fewer than three replicate tests were performed for each group.

**Figure 16:**
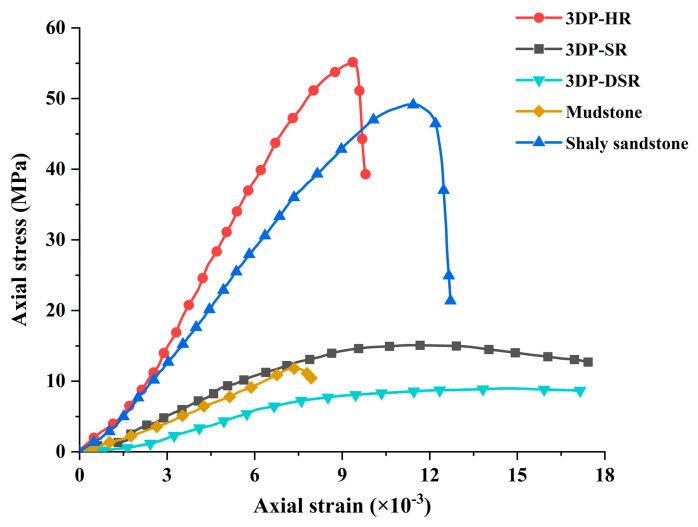
Stress–strain curves of 3DP rock, mudstone [32], and shaly sandstone [30].

The stress–strain curves of 3DP-HR, 3DP-SR, 3DP-DSR, sandstone, and mudstone are shown in Figure 16. The stress–strain curve of 3DP-HR has a relatively steeper slope, whereas that of 3DP-SR exhibits a gentler slope. The rates of axial stress increase and post-peak stress decrease in 3DP-SR are significantly lower than those in 3DP-HR. The stress–strain curve characteristics of 3DP-HR resemble those of sandstone, while those of 3DP-SR are similar to those of mudstone. These findings suggest that the simulating materials used for 3DP-HR and 3DP-SR are appropriate.

**Figure 17:**
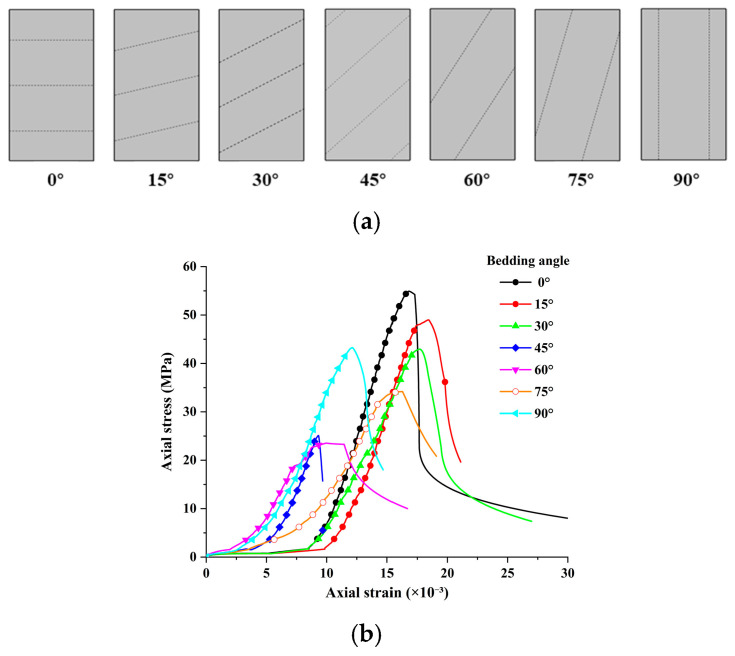
3DP-LHR specimens with different bedding dip angles: (**a**) schematic diagram; (**b**) stress–strain curves.

**Figure 18:**
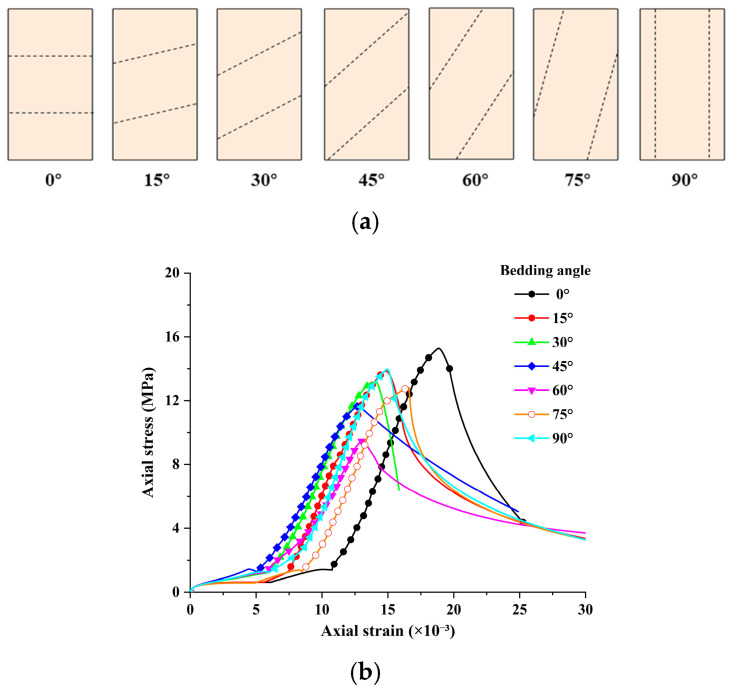
3DP-LSR specimens with different bedding dip angles: (**a**) schematic diagram; (**b**) stress–strain curves.

Schematic diagrams and stress–strain curves of 3DP-LHR and 3DP-LSR specimens with different bedding dip angles are shown in Figure 17 and Figure 18, respectively. In both figures, all stress–strain curves exhibit four common stages: compaction, linear elastic deformation, yielding, and post-peak failure. For the same bedding dip angle, the strain corresponding to the maximum axial stress of 3DP-LSR is generally larger than that of 3DP-LHR, while the rates of axial stress increase and post-peak stress decrease with strain are generally smaller for 3DP-LSR than for 3DP-LHR.

At bedding dip angles of 0° to 30°, the component of axial force perpendicular to the bedding plane is relatively large, and both the matrix and bedding planes are compacted, resulting in a longer compaction stage. In this case, specimen failure is mainly controlled by the matrix, and stress is transmitted primarily through interparticle compression and friction within the matrix. At 45°, the component of axial force perpendicular to the bedding plane decreases significantly, while the component parallel to the bedding plane increases substantially. Under this condition, specimen failure is jointly controlled by the matrix and bedding planes. At 60° to 75°, the component of axial force parallel to the bedding plane increases further, and specimen failure is primarily controlled by the bedding planes, with the load mainly sustained by friction and mechanical interlocking along the bedding planes. At 90°, both the matrix and the bedding planes become vertically continuous, and specimen failure is jointly controlled by the matrix and bedding planes. The matrix serves as the main load-bearing component, while fracture development along the bedding planes weakens its load-bearing capacity. For bedding dip angles of 45° to 90°, the compaction stage is generally relatively short. As the bedding dip angle increases, the bedding planes become progressively more parallel to the axial loading direction and therefore are not fully compacted during loading.

### 5.2. Anisotropy of Mechanical Parameters

**Figure 19:**
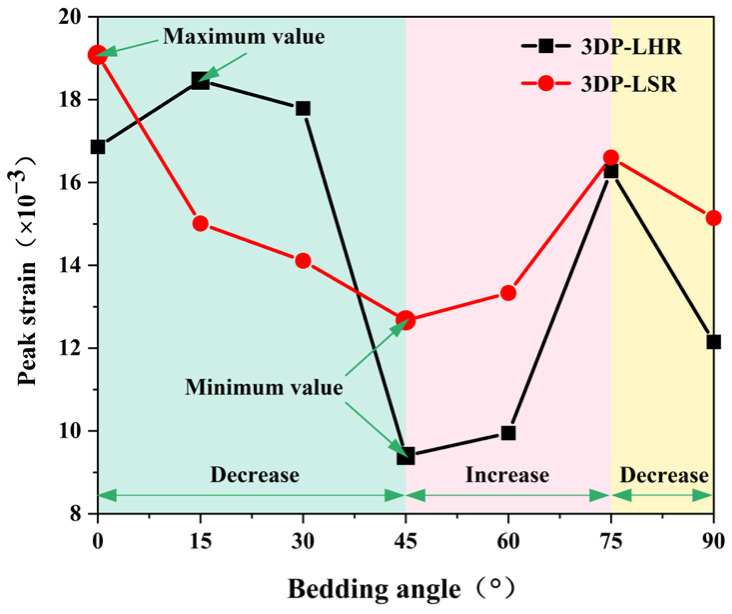
Strain at peak stress of 3DP-LHR and 3DP-LSR specimens as a function of bedding dip angle.

Based on the representative stress–strain curves presented in Figure 17 and Figure 18, the variation in strain at peak stress (SPS) of the 3DP layered rock specimens with bedding dip angle was obtained, as illustrated in Figure 19. The minimum SPS values consistently occur at 45°. Axial displacement results from both axial compression and sliding along the bedding planes. From 0° to 45°, the axial compression component of displacement gradually decreases, whereas the component induced by bedding plane sliding progressively increases. The combined effect of these two components causes the total axial displacement to continuously decrease, reaching its minimum at 45°. At 60°, shear slip failure occurs before sufficient compaction, further reducing the axial compression displacement compared with that at 45°. However, the displacement caused by bedding plane sliding becomes larger, resulting in a slightly higher SPS at 60° than at 45°. At 75°, the intermediate rock layers are fully interconnected, with a large cross-sectional area at the specimen ends. As a result, the axial compression displacement increases significantly, while the displacement associated with bedding plane sliding remains substantial. In addition, eccentric deformation occurs, leading to a marked increase in SPS from 60° to 75°. At 90°, the specimen mainly exhibits axial compression displacement, resulting in a renewed decrease in SPS.

**Figure 20:**
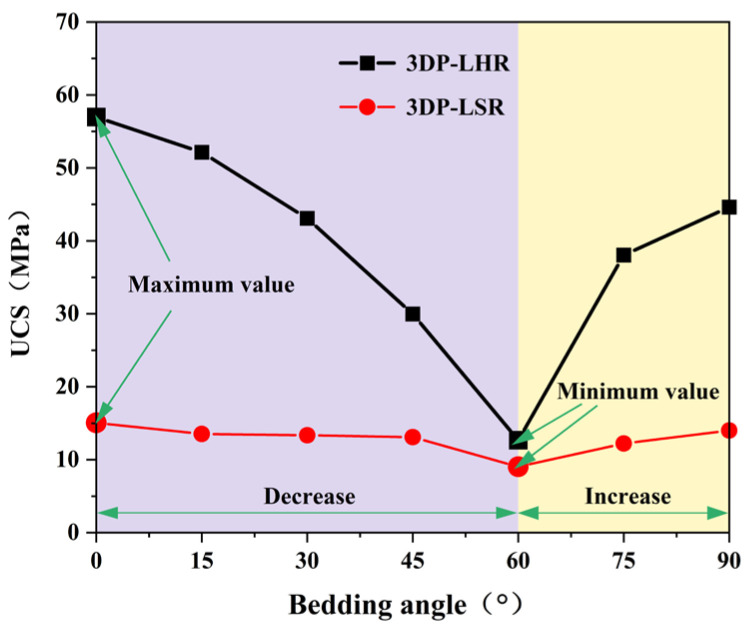
UCS of 3DP-LHR and 3DP-LSR specimens as a function of bedding dip angle.

**Figure 21:**
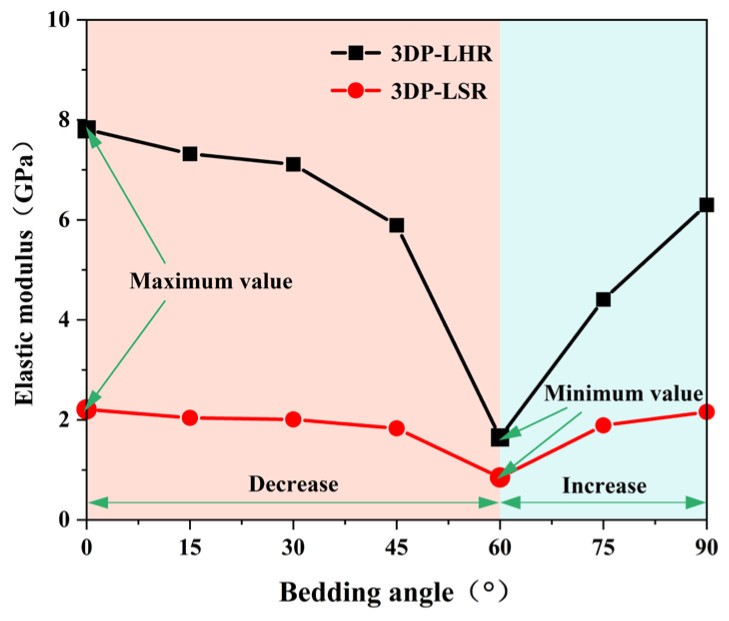
Elastic modulus of 3DP-LHR and 3DP-LSR specimens as a function of bedding dip angle.

**Figure 22:**
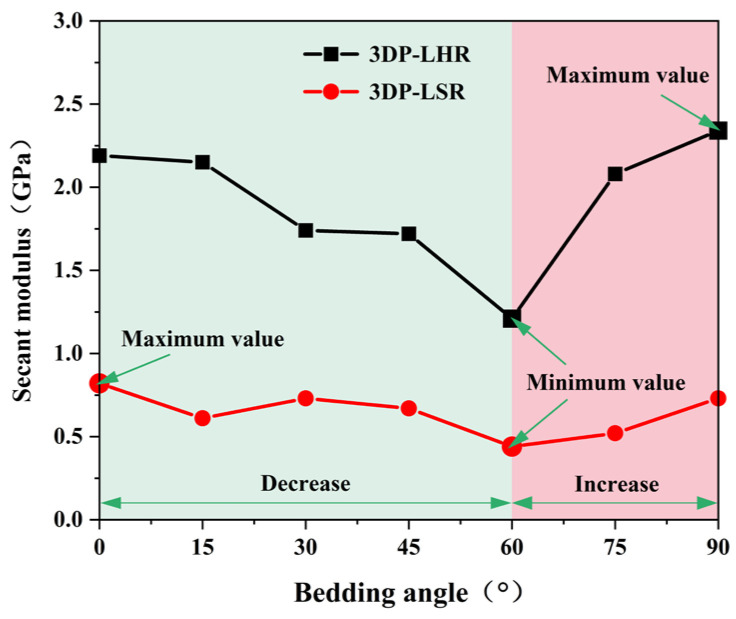
Secant modulus of 3DP-LHR and 3DP-LSR specimens as a function of bedding dip angle.

At least three UCTs were performed on 3DP-LHR and 3DP-LSR specimens at each of the seven bedding dip angles. After excluding data points that deviated from the arithmetic mean by more than ±10%, the mean values of the remaining valid data were calculated and are plotted in Figure 20, Figure 21 and Figure 22. The UCS, elastic modulus, and secant modulus all exhibit a trend of first decreasing and then increasing with increasing bedding dip angle, reaching their minimum values at a bedding dip angle of 60°, which is consistent with previous studies [39,41,42,43]. All three parameters display the greatest variation and highest sensitivity near 60°. This is because, as the dip angle approaches 60°, the ratio of shear stress to normal stress resolved on the bedding plane becomes most favorable for sliding. Under this condition, shear failure along the weak bedding plane and compressive failure of the matrix jointly compete and interact, such that even a small change in dip angle can trigger a shift in failure mode and a sharp fluctuation in load-bearing capacity.

A key finding is that both 3DP-LHR and 3DP-LSR exhibit generally higher mechanical parameters at 0° than at 90°, although opposite trends have also been reported in the literature. This divergence is mainly governed by the bedding-plane properties. When bedding planes are weakly cemented and defect rich, as in 3DP specimens, normal compression at 0° tends to close interfacial defects, and failure is mainly controlled by the matrix, yielding higher strength. At 90°, lateral tensile deformation induced by Poisson’s effect opens these weak interfaces, inducing premature splitting failure and a sharp reduction in strength. Conversely, when the bedding planes are relatively strong, this trend can be reversed. The inherent interfacial porosity and weak cementation in 3DP specimens therefore explain the observed strength hierarchy. Moreover, the preferred fiber orientation [44] further amplifies this difference: at 0°, fibers lie approximately perpendicular to the loading direction, providing lateral confinement; at 90°, they are aligned nearly parallel to the load, offering negligible resistance to lateral cracking. The coupling effect of bed-ding-plane defects and fiber orientation is therefore the root cause of the mechanical contrast, resulting in more favorable mechanical behavior at the 0° orientation.

Figure 20, Figure 21 and Figure 22 also show that the variation in mechanical parameters with bedding dip angle is markedly less pronounced for 3DP-LSR than for 3DP-LHR, with a much smaller maximum-to-minimum ratio. The weaker interparticle cementation in 3DP-LSR facilitates plastic deformation and promotes a more uniform redistribution of stress and strain energy, thereby mitigating the bedding-induced mechanical contrast. In contrast, the stronger cementation in 3DP-LHR more firmly constrains the particles in their initial positions and orientations, suppressing plastic deformation of the matrix. Consequently, bedding-plane defects cannot be effectively accommodated by the matrix, rendering the material highly defect sensitive and further accentuating the mechanical weakness along the bedding planes. When the loading direction changes, the failure mode gradually shifts from tension–shear fracture across the bedding planes to brittle slip or tensile cracking along them; thus, bedding defects become preferred crack propagation paths, leading to a much stronger angular dependence of the mechanical parameters.

### 5.3. Fracture Characteristic Analysis

Failure images and failure modes of 3DP-LHR and 3DP-LSR are shown in Table 7. Under uniaxial compression, the failure modes of the 3DP layered rocks also exhibit pronounced anisotropic characteristics. The primary fracture characteristics of 3DP-LHR, 3DP-LSR, and the layered sandstone presented in ref. [39] are generally similar, although some differences exist in local crack features and failure modes.

(1) At bedding dip angles of 0–30°, the bedding planes are subjected to normal compression, and the associated defects tend to close; therefore, failure is predominantly governed by the matrix. At 0°, both 3DP-LHR and layered sandstone undergo splitting tensile failure that cuts through the matrix and bedding planes, with axial loading suppressing the mechanical degradation of the weak planes. 3DP-LSR also fails mainly through the matrix but develops more shear cracks, exhibiting combined tension–shear failure due to weak interparticle cementation and local plastic flow. Layered sandstone, with better cementation and denser bedding planes, fractures preferentially through the matrix, with only local bedding-parallel propagation. At 15° and 30°, all three types undergo combined tension–shear failure across the matrix and bedding planes while remaining matrix dominated, indicating that the normal stress acting on the bedding planes at low dip angles is sufficient to suppress the activation of interfacial defects.

(2) At 45°, interfacial defects begin to be activated, and failure is jointly governed by the matrix and bedding planes. 3DP-LHR exhibits both shear sliding along bedding planes and tensile fracture across them, indicating that the interfacial shear strength is approaching a critical state, while the matrix still retains a certain locking effect. Layered sandstone, with stronger bedding cementation, fractures mainly across bedding planes with limited bedding-plane damage, demonstrating the failure-delaying effect of higher interfacial strength. In contrast, 3DP-LSR shows no macroscopic tensile crack and fails by shear sliding along and partially cutting across bedding planes; matrix plastic deformation dissipates tensile stress, forcing failure to rely predominantly on shear sliding.

(3) At bedding dip angles of 60–75°, failure is fully dominated by the bedding planes: both 3DP-LHR and layered sandstone fail by shear sliding along the bedding planes. Although 3DP-LSR also fails primarily by bedding-parallel sliding, tension–shear cracks simultaneously develop in its matrix, reflecting the inability of the weakly cemented matrix to fully confine stress concentration to the interfaces. Strong cementation tends to concentrate stress at bedding defects, promoting brittle sliding, whereas weak cementation allows stress redistribution through plastic yielding.

(4) At 90°, the bedding planes become planes of tensile weakness, and differences in bedding-plane density and continuity lead to distinct failure responses. All three specimen types exhibit tensile splitting along the bedding planes. However, no shear cracks are observed in the sandstone matrix, whereas shear cracks develop in the matrices of both 3DP-LHR and 3DP-LSR. This can be attributed to the densely developed bedding planes in sandstone: splitting cracks propagate along numerous parallel weak planes, producing a stress-partitioning effect that relieves stress in the adjacent matrix and prevents it from reaching the shear-yielding condition. In contrast, the 3DP specimens have more sparsely distributed bedding planes, which are weakly cemented cold joints. After cracks propagate along the bedding planes, matrix bridging zones remain, and secondary shear cracks are generated under coupled tensile–shear stress. A similar phenomenon has been reported in shale with densely developed bedding [42].

Overall, the failure mode evolution of the 3DP layered rocks with bedding dip angle is consistent with that of natural layered rocks. From 0° to 90°, the primary fracture location shifts from the matrix to the bedding planes and eventually to the combined failure of both components, matching the initial decrease and subsequent increase in the mechanical parameters. Owing to its high bedding density and strong cementation, layered sandstone exhibits the weakest shear plastic deformation; 3DP-LSR displays the strongest shear fracturing because of weak interparticle cementation, while 3DP-LHR shows an intermediate response. These phenomena are fundamentally driven by the evolving competition between bedding-plane defects and matrix cementation.

## 6. Discussion

A noteworthy and counterintuitive phenomenon is observed: although the porosities of 3DP-SR and 3DP-DSR are much higher than that of 3DP-HR, their densities are also higher. This can be attributed to the fact that the density-increasing effect of high-density barite powder outweighs the density-reducing effect of rosin-induced foaming. It should be noted, however, that the air-entraining action of rosin is sensitive to mixing speed, temperature, and cement type, which may introduce variability into the resulting porosity and pore structure. Moreover, the resulting combination of high porosity and high cementitious content warrants careful assessment in terms of long-term durability, particularly with regard to shrinkage and potential strength retrogression. With due consideration of these factors, the simultaneous incorporation of rosin and barite powder from the solid waste offers a potential route to developing unconventional cement-based materials that combine high porosity with high density, which may be of interest for applications such as radiation shielding or sound insulation.

The method developed here for fabricating layered rock models represents a new attempt, but it still has several limitations. First, the layer-by-layer deposition process inherent to 3DP unavoidably introduces defects such as interlayer cold joints and micropores, whose meso-structural characteristics and mechanical properties differ from those of natural bedding planes formed over geological time. The fact that our results nonetheless align well with published data suggests that the 3DP rock models can capture the essential mechanical response of layered rocks, although accurate quantitative prediction still requires explicit consideration of these inherent printing-induced defects. Second, even with strict adherence to testing standards and a minimum of three replicates per group, some data scatter persisted; therefore, the overall trends were checked against the literature and found to be consistent. Third, the simulation of bedding planes remains relatively preliminary: parameters such as roughness, thickness, cohesion, and internal friction angle have not been quantified or varied independently, so their individual contributions could not be isolated. Future studies should employ multi-material, multi-nozzle 3DP platforms combined with subtractive processes such as milling so that these bedding-plane characteristics can be tuned independently. This would enable the production of models with well-defined properties and facilitate systematic investigation of the underlying mechanical mechanisms.

## 7. Conclusions

In this study, rock and layered rock models were fabricated using cement-based 3DP technology. Based on UCTs, P-wave velocity measurements, and CT scanning, the inherent anisotropy of the 3DP rock was quantitatively evaluated, and the mechanical anisotropy and failure modes were compared between two types of 3DP layered rocks and natural layered sandstone. The main findings are as follows:

(1) The key physical and mechanical parameters of the developed cement-based 3DP material, including UCS, elastic modulus, P-wave velocity, and CT number, are close to those of natural rocks. This indicates that, even without deliberately matching porosity, this material can be feasibly used to simulate natural rocks.

(2) For this 3DP rock, the variations in average UCS, average P-wave velocity, and CT number along the X-, Y-, and Z-directions are all within 6%. These directional variations are smaller than those observed in sandstone and some natural rocks, suggesting that the inherent anisotropy of the 3DP rock has only a minor influence on the test results.

(3) For the 3DP layered rocks, the UCS, elastic modulus, and secant modulus first decrease and then increase with increasing bedding dip angle, reaching their minimum values at 60°. This trend aligns with the typical behavior reported in existing studies on layered rocks.

(4) Under uniaxial compression, the dominant failure characteristics of the 3DP layered rocks are similar to those of layered sandstone at the same bedding dip angle. At 0°, splitting tensile failure dominates. At 15° and 30°, combined tension–shear failure occurs. At 45°, shear sliding along bedding planes is accompanied by local tensile failure of the matrix. At 60° and 75°, pure shear sliding along bedding planes dominates. At 90°, splitting tensile failure along bedding planes occurs together with tension–shear failure in the matrix. From 0° to 90°, the primary fracture location in layered rocks shifts from the matrix to the bedding planes and eventually to the combined failure of both components.

This study demonstrates the feasibility of using cement-based 3DP to fabricate layered rock models from three perspectives: the mechanical properties of the simulated material, the inherent anisotropy of the 3DP itself, and the failure modes. It provides a new approach for physical and mechanical experiments on layered rocks, featuring low cost, low variability, and high repeatability.

## Figures and Tables

**Table 1:** Dosages of each component in the mixture proportion for the hard rock-simulating material.

Component	425 Portland Cement	Sulfoaluminum Cement	River Sand	Water Reducer	Defoamer	Emulsion Powder	Cellulose Ether	Polypropylene Fiber	Water
Quality	9.5 kg	0.5 kg	10.0 kg	20.0 g	10.0 g	60.0 g	6.0 g	20.0 g	3.2 kg

**Table 2:** Comparison of UCS and elastic modulus between sandstone and 3DP-HR.

Category	UCS/MPa	Elastic Modulus/GPa
Sandstone [29]	36.94	6.26
Sandstone [30]	49.18	5.31
Sandstone [31]	49.92	11.79
3DP-HR	47.55~54.86	6.99~8.04

**Table 3:** Dosages of each component in the mixture proportions for the soft rock-simulating material and the degraded soft rock-simulating material.

Category	Solid Waste/kg	Cement/kg	River Sand/kg	Water Reducer/g	Defoamer/g	Emulsion Powder/g	Polypropylene Fiber/g	Cellulose Ether/g	Water/kg
Soft rock simulant	7.5	3	5	18.29	18.29	0	20.03	6.62	2
Degraded soft rock simulant	9	3	5	20.68	20.68	0	22.65	7.49	2.27

**Table 4:** Comparison of UCS and elastic modulus among mudstone, 3DP-SR, and 3DP-DSR.

Category	Confining Pressure/MPa	UCS/MPa	Elastic Modulus/GPa
Mudstone [32]	0	11.96	1.75
Mudstone [33]	1	19.79	3.37
3DP-SR	0	13.28–14.39	1.46–1.72
3DP-DSR	0	8.43–10.60	1.09–1.53

**Table 5:** Density, porosity, and pore size distribution of 3DP-HR, 3DP-SR, and 3DP-DSR specimens.

Category	Air-Dried Density/g·cm^−3^	Saturated Density/g·cm^−3^	Porosity ——	Pore Size Distribution (μm)				
				0–0.1	0.1–0.4	0.4–1	1–2.5	2.5–10
3DP-HR	2.136	2.162	2.48%	2.362%	0.083%	0.026%	0.0006%	0.0103%
3DP-SR	2.247	2.369	7.64%	7.411%	0.056%	0.015%	0.0963%	0.0459%
3DP-DSR	2.182	2.343	8.66%	8.119%	0.380%	0.006%	0.0899%	0.0546%

**Table 6:** Main mechanical parameters of 3DP-HR, 3DP-SR, and 3DP-DSR specimens.

Category	UCS/MPa	Elastic Modulus/GPa	Tensile Strength/MPa	Cohesion/MPa	Internal Friction Angle/°
3DP-HR (Z-direction)	47.55–54.86	6.99–8.04	3.86	7.28	51.92
3DP-HR (X-direction)	50.54–56.50	7.07–7.98	——	——	——
3DP-HR (Y-direction)	53.24–59.51	7.66–8.89	——	——	——
3DP-SR	13.28–14.39	1.46–1.72	1.74	2.21	48.73
3DP-DSR	8.43–10.60	1.09–1.53	1.53	1.81	49.71

**Table 7:** Failure images, sketches, and failure modes of 3DP-LHR and 3DP-LSR specimens at different bedding dip angles.

Layer Angle	3DP-LHR	3DP-LHR Sketch	3DP-LSR	3DP-LSR Sketch
0°	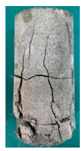	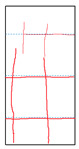	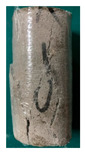	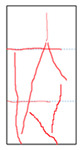
Failure mode	Splitting tensile fracture through matrix and bedding plane		Combined tensile–shear failure across matrix and bedding plane	
15°	** 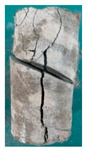 **	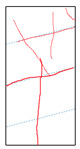	** 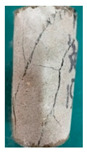 **	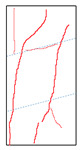
30°	** 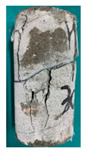 **	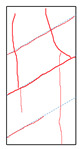	** 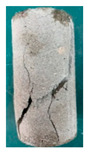 **	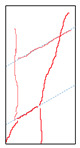
Failure mode	Combined tensile–shear failure across matrix and bedding plane		Combined tensile–shear failure across matrix and bedding plane	
45°	** 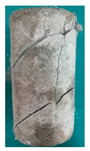 **	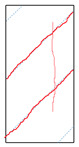	** 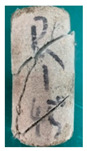 **	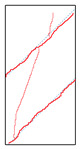
Failure mode	Sliding failure along bedding plane and tensile fracture across bedding plane		Sliding failure along bedding plane and local cross bedding plane (oblique crossing)	
60°	** 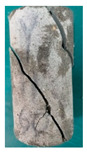 **	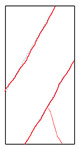	** 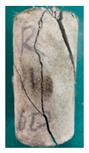 **	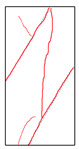
75°	** 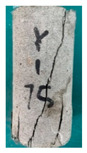 **	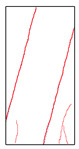	** 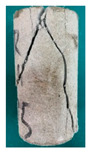 **	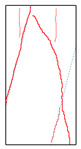
Failure mode	Sliding failure along bedding plane		Sliding failure along bedding plane and combined tensile–shear failure in the matrix	
90°	** 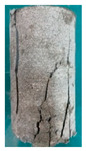 **	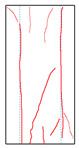	** 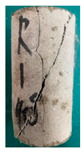 **	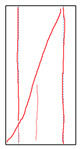
Failure mode	Tensile split along bedding plane and sliding failure in the matrix		Tensile split along bedding plane and sliding failure across bedding plane (oblique crossing)	

## Data Availability

The original contributions presented in this study are included in the article. Further inquiries can be directed to the corresponding author.
